# Habitat Preferences of Butterflies in the Bumbuna Forest, Northern Sierra Leone

**DOI:** 10.1673/031.008.6401

**Published:** 2008-10-24

**Authors:** Abu James Sundufu, Rashida Dumbuya

**Affiliations:** Department of Biological Sciences, School of Environmental Sciences, Njala University, Sierra Leone

**Keywords:** species richness, species diversity

## Abstract

The habitat preferences of the butterfly fauna were studied in the Bumbuna Forest Reserve in northern Sierra Leone. The intact forest reserve and a secondary forest regrowth, disturbed as a result of slash-and-burn agriculture, were compared to savanna habitats. Of the 290 specimens collected, 195 butterfly species were included, of which significant proportion were Nymphalidae. Of the 147 forest species, 111 (75.5%) showed preferences for the forest habitats, while 70 (47.6%) and 34 (23.1%) preferred disturbed and savannah habitats, respectively. Numerically, a comparable proportion of savannah species were recorded in the 18 disturbed (73.9%) and 16 savannah habitats (63.2%). Accumulated species richness and diversity indices were lower in the disturbed habitats compared to the forest reserve, but lowest in the savanna habitats. However, a large proportion of forest species, especially those with either a more restricted geographic range or species for which no information on geographic distribution was available, were exclusively captured in the forest patches. The survey indicated the presence of a rich butterfly fauna, which should be systematically collected for further research and study in order to build a good taxonomic database for Sierra Leone.

## Introduction

Tropical forest ecosystems are under enormous pressure all over the world. Many forest areas in the tropics may only persist as production areas (Brown 1997, Hunter 1999), and pressure on unprotected forests is very likely to escalate (Terborgh 1999, Lewis 2000). Despite their generally recognized importance for global diversity (Sutton and Collins 1991; World Conservation Monitoring Centre 1992), no more than 4% of tropical forests are situated within the boundaries of reserves or national parks (Whitmore and Sayer 1992). Even the best protected areas might not be adequate to maintain the original ecosystems because of their small size and difficult political and social circumstances (Terborgh 1999). Although the magnitude of biodiversity present on Earth is largely unknown (Dobson 1995) and its estimates remain highly controversial (May 1990; Stork 1988), it is generally accepted that much, if not most, of the global diversity in terms of numbers of species is represented by arthropods inhabiting tropical rainforests (Wilson 1988). Still, few data are available about the effects of forest disturbance upon these species-rich insect faunas (Klein 1989; Holloway et al. 1992; Eggleton et al. 1995;). Butterflies, however, are comparatively well studied. Butterfly species composition in disturbed and undisturbed forests has been investigated for example in Southeast Asia (Spitzer et al. 1993; Hill et al. 1995; Beck and Schulze 2000), Madagascar (Kremen 1992), and the Neotropics (Lovejoy et al. 1986; Brown 1991; Sparrow et al. 1994; DeVries et al. 1997; Wood and Gillman 1998). Several studies showed that low disturbance levels have a positive effect on diversity and abundance of rainforest butterflies (Lovejoy et al. 1986; Brown 1991; Sparrow et al. 1994; Wood and Gillman 1998). These results are in accordance with the intermediate disturbance theory (Connell 1978) and have parallels in temperate forest habitats, where forest management providing a large range of shade levels has been found to increase the number of habitats suitable to different butterfly species (Warren 1985). In contrast, other studies indicate adverse effects of disturbance on tropical butterfly communities (Thomas 1991; Spitzer et al. 1993, 1997; Kremen 1994; Hill et al. 1995, 2001; Brown 1997; Hill and Hamer 1998; Hamer and Hill 2000, Lewis 2000; Fermon et al. 2000, 2001), indicating an increase in diversity and/or abundance of widespread, common butterfly species and a decline in restricted range species after disturbance. Although deforestation rates are highest in several West African regions, little is known about the effects of forest disturbance on afrotropical butterflies (Larsen 1995a). In Madagascar, disturbed forest habitats and edges were equally found to be richer in species than undisturbed areas (Kremen 1992, 1994). Also in southern Nigeria, Larsen et al. (1979) found a surprisingly rich butterfly fauna in mixed secondary growth within the rainforest zone.

In south-central Benin, overall butterfly species richness was higher in clearings than closed forest, however, a high proportion of forest understorey species with a restricted geographic range were exclusively captured in closed forest patches (Fermon et al. 2001). Whereas there is still much work to do to describe the biodiversity of unmodified forest systems, questions concerning ecologically sound management plans cannot be answered without proper assessment in managed forest areas. Considering the high deforestation rates and the fact that a combination of ecology and economy is often the only strategy to protect the rich rainforest biodiversity in many developing countries (Brown 1997), these assessment studies will become increasingly important. The present study mainly documents habitat specificity and diversity of butterflies in the disturbed Bumbuna Forest Reserve in northern Sierra Leone. The study took place within the framework of an Environmental Impact Assessment Survey (Bumbuna Hydroelectric Project) and the data reported here will be included in this survey (TB Larsen in preparation).

## Materials and Methods

### Study area

Sierra Leone is located on the Atlantic Coast of West Africa, and lies at the western end of the Upper Guinea Forest Block. It is one of the more severely deforested countries in the region (Barrie 2002). Bumbuna is located in the Northern Province along the valley of river Seli. It is a Headquarter town in the Kalansogoia Chiefdom with a total of 65 villages. The total population is 1,700 in about 400 households, the majority of who are farmers. The climate in the study area reflects the general climatic pattern in Sierra Leone, which can be classified as a tropical savannah climate with a distinct tropical wet and dry season. The wet season starts in May and ends in October. Thunderstorms, accompanied by heavy rains, characterize both the start and end of this period. The dry season (November-April) is usually interspersed with the harmattan, a dry dust-laden wind blowing from the Sahara, which occurs between late December and early February, bringing low humidity and relatively cool night temperatures. Highest temperatures are in March with 35°C. Rainfall in the area indicates an average annual precipitation of 2635 mm with maximum in August of 600 mm. The vegetation of the study area is a forest-savannah mosaic type consisting of patches of closed forest communities and serial stages interspersed within savannah woodlands. Closed moist forest regrowth and thicket (secondary forests) and savannah woodland are the three major plant communities occurring predominantly in the area. Three other plant communities present to a more limited extent were (i) fringing forests along rivers and streams (gallery forests), (ii) inland valley swamps (cultivated and natural) and (iii) upland grassland and/or sedges on granite outcrops.

### Study site

**Figure 1.**
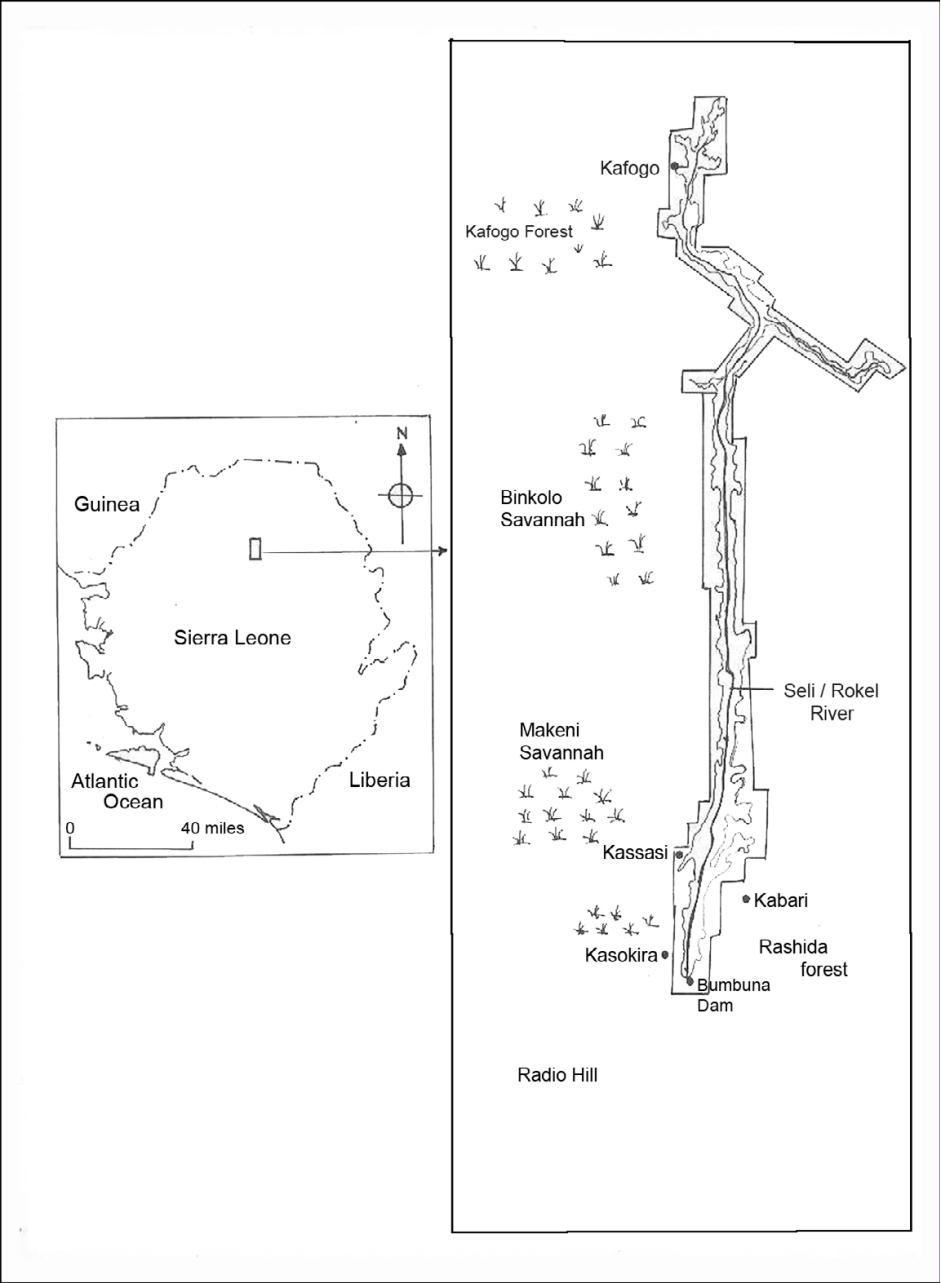
Map of the study sites showing two types of forests (Rashida forest, Radio Hill), three types of savannahs (Kasokira road, savannah to Makeni, savannah-Binkolo to Kafogo), and three types of disturbed habitats (Road leading to Kasassi, Kabari village, Kafogo forest).

Many butterflies are localized or restricted to specific habitat types. For this survey, sample sites included: two types of forests (Rashida forest, Radio Hill), three types of savannahs (Kasokira road, savannah to Makeni, savannah-Binkolo to Kafogo), and three types of disturbed habitats (Road leading to Kasassi, Kabari village, Kafogo forest) (Figure 1). The forest habitats are not virgin forests but rather high forests with vegetation cover mostly canopy and sub-canopy. Although, it had not been disturbed for at least 25 years, it is presently under going felling. The 4 sq km Rashida Forest is located in the upper valley and on the right hand of the dam. This forest will be drowned in water upon the inundation of the Bumbuna Hydroelectric Project. Radio Hill is smaller and located on the route to the residential quarters. In the savannah habitats, the vegetation is predominantly grass and small-scale farming is practiced. The disturbed habitats are farm bush habitats with about 3–5 years fallow period, which has decreased by 1 year over this period. Even when recovering from activities like large-scale slash-and-burn farming and logging, small-scale farming for the cultivation of vegetables by local people continues.

### Fruit trapping

Traps used were basically as described by DeVries (1987, 1988) and Mühlenberg (1993) and the bait was a fermented banana. Species from 4 out of the 7 African Nympahlidae subfamilies (Libytheinae, Danainae, Satyrinae, Charaxinae, Apaturinae, Nymphalinae and Acraeinae) use fermenting fruit as a resource, including Nymphalinae, Satyrinae, Charaxinae and Apaturinae. In Africa, the following Nymphalinae genera feed consistently on fermenting fruit: Euphaedra, Bebearia, Euriphene, Euryphura, Cymothoe, Pseudacraea, Euptera and Pseudathyma (Larsen 1994a). Some other Nymphalinae such as Hypolimnas, Salamis and Antanartia are found on both flowers and fruits. Most tropical Satyrinae, especially within the Bicyclus and Gnophodes, are exclusively fruit-feeders. The Charaxinae and Apturinae are attracted to both fruit and rotting animal matter and faeces. The baited traps were installed 1.0 m above the ground within the study site. Three traps were situated in each of 3 habitats: the understorey of mature forest patches, disturbed forest and savanna habitats. Traps were checked every 24 h and baited with rotting banana, mango and animal faeces. The traps were regularly moved to cover most of the collecting area.

### Walk-and-capture

Walk-and-capture transect routes of 1 km each were surveyed during 2 weeks. Three transects were walked between 09:00 and 12:00 in the morning and between 15:00 and 17:00 in the afternoon under sunny weather conditions, each for a duration of 2 hours. Three transects were situated in each of the three habitats: undisturbed forest, disturbed forest and savannah. A total of 14 person-hours were obtained for each habitat. All butterflies seen 2.5m either side of the transect route and up to 5m in front were trapped or released after marking when positive identification was possible (Pollard 1977; Hill et al. 1995).

### Butterfly identification and geographic range classification

Butterflies collected were identified using ‘Butterflies of West Africa’ (Larsen 2005). Habitat associations (preference for certain forest types) and geographic distributions were adopted from Belcastro and Larsen (2006).

### Calculation of community parameter

Butterfly diversity was estimated using the following estimators: Shannon-Weiner (H') diversity index (Magurran 1988) and Evenness (J'). The mean number of individuals in each family was calculated and mean separation done using the Least Significant Difference (LSD) test (SAS 1998). Differences in species richness were tested among the forest, disturbed and savannah habitats comparing number of species (S), Shannon-Weiner (H') diversity and Eveness (J') with Kruskal-Wallis ANOVA. Kruskal's gamma rank correlation coefficient (*γ*) was used to analyze the relationship between geographic range and habitat specificity (Statsoft 1995).

## Results and Discussion

### Species richness

A total of 195 butterfly species were recorded within the Bumbuna Forest Reserve during the present study (see Appendix). The Lycaenidae (19.00 ± 1.45) and Hesperiidae (13.67 ± 4.67) are under- represented, constituting only 20% and 14% of the total butterfly fauna. Due to the focus on the fruit-feeding butterfly community, the Nymphalidae family is significantly (F_1,4_ = 18.48, P < 0.05; 49.33 ± 9.60) represented and comprises 51% of the butterfly fauna listed: members of the subfamily Limenitidinae (35 species) with *Euhaedra*, *Neptis* and *Bebearia* as important forest under-storey genera, Charaxinae (16 species) with *Charaxes* and *Palla* as dominant genera and Satyrinae (14 species) with the genera *Bicyclus.* The Papilionidae (6.67 ± 2.19) and Pieridae (7.67 ± 2.16) make up 7% and 8%, respectively of the total records. No species of the family Riodinidae was recorded.

The total butterfly abundances differ significantly among the three areas (2-way ANOVA, F1,2 = 3.83, p < 0.05), with the highest number captured from the forest habitat. The Shannon-Weiner diversity indices calculated for each sample were significantly higher for both the forest and the disturbed habitats compared to the savannah habitat (Kruskal-Wallis ANOVA, H = 27.02 and P < 0.05) and no significant difference could be found for evenness (Table 1).

Other surveys of butterflies have been conducted in the Bumbuna Forest yielding totals different from this survey. Larsen in his recent survey in May 2006, recorded 313 butterfly species during one month (including the 195 included in the current survey), while Belcastro (1990b, 1986a,b) made collections, though not regularly, and recorded additional 131 species. Thus 444 species are known from the Bumbuna area. According to TB Larsen (personal comm.) another 50 species or so should occur for a total of about 500 in all. According to this estimate, the Bumbuna Forest would comprise 50% of all West African species (West of the Dahomey Gap).

**Table 1.**
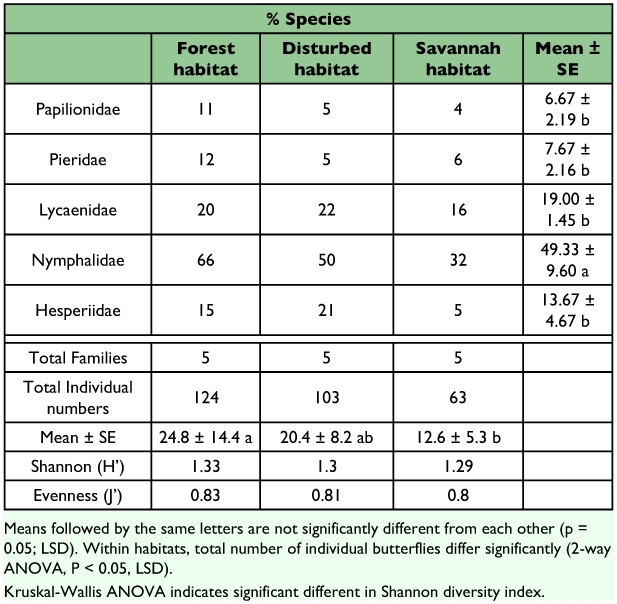
Summary of butterfly individuals captured by family and habitat type.

The estimated total species richness is comparable with that of the roughly 21,600 ha large Bossematié Forest (also with about 500 species in total) (Larsen 1994b, 1995a). The actual number of species recorded in this study represent only three-fourths of those recorded in Bossematié Forest.

### Ecological composition

Most African butterflies tend to be restricted to one or a limited number of ecological zones and are found in specific habitats (Larsen 1995a). For example, there is a very large difference in total species between fauna of the forest and the northern Sudan savannas, which are separated by the Guinea savanna (Larsen and Mei 1998). The butterfly fauna of West Africa (west of the Dahomey Gap) consist of approximately 1000 species (Larsen 2005).

The species recorded in the Bumbuna Forest thus amount to 19.6% of all butterflies recorded in West Africa (Table 2). Although slash-and-burn agriculture has resulted in a mosaic of forest and disturbed habitats, the overall ecological conditions of the Bumbuna Forest still appear to meet the habitat requirements of a large number of forest species.

However, the number of forest butterflies species recorded in this study accounts for only 18% of all West African forest species. By contrast, approximately 66% of all West African ubiquitous species were recorded. Only 18% of all savanna species were recorded, which is less than might have been expected. Both ubiquitous and savanna species constitute approximately one fourth of the total number of butterflies sampled in the Bumbuna Forest.

147 (75.4%) of the recorded butterfly species belong to the category of forest species (Table 2), species centered on closed forests that do not usually colonize savanna or other open habitats (Larsen 1994b, 1998; Emmel and Larsen 1997). Only a small fraction are either ubiquitous species (9.8%) or habitat specialists linked to swampy zones (1.5%) or belong to the savanna butterfly community (13.3%). Almost half of all true forest species are species found generally distributed in all forest types, whereas 26% are centered on the moist semi-deciduous forests. Despite the significant number of true forest species, only 18% of all West African forest species have been recorded in the present study.

**Table 2.**
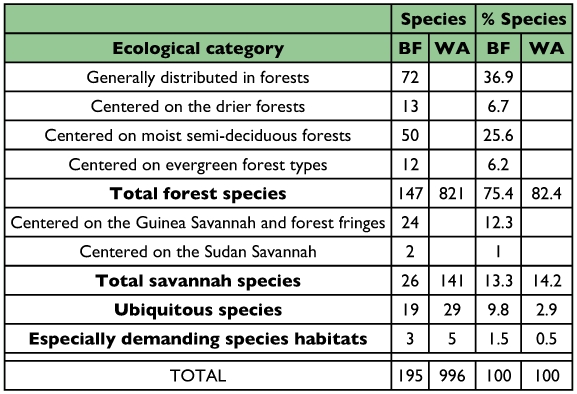
Number of butterfly species collected in the Bumbuna Forest (BF) in the present study by ecological category (Belcastro and Larsen 2006), compared with the total fauna of West Africa (WA) west of the Dahomey Gap (data on the West African butterfly fauna after Larsen and Mei 1998).

### Habitat preference and geographic distribution

Tables 3 and 4 show the number of species recorded during this study in the Bumbuna Forest and do not include the additional species listed by Larsen (2006). As expected, 76% of species classified as forest (Larsen 2006) were collected in the Bumbuna Forest Reserve, while 48% of forest species were collected in farm bush. This suggests that the forest butterflies were largely “robust” species that can survive in farm-bush and small bits of forests and village fruit and sacred groves. Twenty-three percent (23%) of forest species were even found in savanna, mainly in small gallery forests along streams.

**Table 3.**
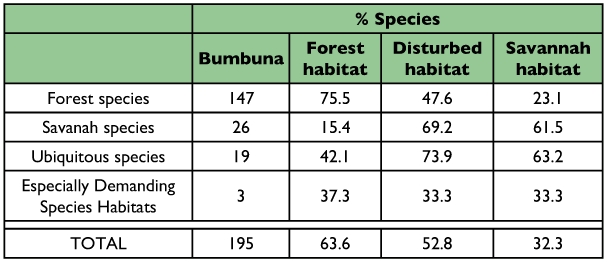
Number of butterfly species per ecological category (Belcastro and Larsen 2006) recorded in Bumbuna during the present study, northern Sierra Leone, and percentage of species exclusively recorded in either forest, disturbed or savannah habitats within Bumbuna.

By contrast, the largest proportion of savanna species were found in disturbed habitats (69%). A comparable proportion was recorded in the savanna habitat (62%), which was not surprising. However, less than one fourth were recorded in forest habitats.

The proportion of species present in the forest, disturbed and savanna habitats within the Bumbuna Forest Reserve, classified according to their geographic range (Belcastro and Larsen 2006), also show a clear pattern (Table 4). A significant negative correlation between geographic range and habitat specificity was visible in the 195 species captured (gamma rank correlation for multiple ties, *γ* = -0.2737, n = 195 spp., P < 0.001). The proportion of species present in forest appears to increase with decreasing geographic range. Only 40% of the species recorded in forest belong to the most widespread group of species, as compared to 61.8% in disturbed and 52.7% in savanna habitats.

**Table 4.**
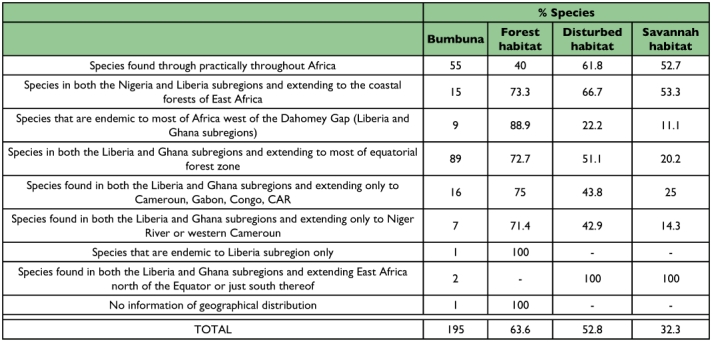
Number of butterfly species by geographic area (Belcastro and Larsen 2006) recorded in Bumbuna, Northern Sierra Leone, and percentage of species exclusively recorded in either forest, disturbed or savannah habitats within Bumbuna.

Overall species richness in the forest is comparatively the high. It might therefore, be expected that species with a smaller geographic range will thrive in restricted habitats having specific requirements within the Bumbuna Forest Reserve. This emphasizes their importance for maintaining biodiversity on a regional scale. Similar patterns have been found for other West African (Fermon et al. 2000) and South East Asian (Hamer et al. 1997; Hill et al. 1995; Spitzer et al. 1993) forest butterflies. Species with a restricted geographic distribution appear to be more sensitive to human disturbance and forest structure changes than widespread species.

## Figures and Tables

**Appendix.**
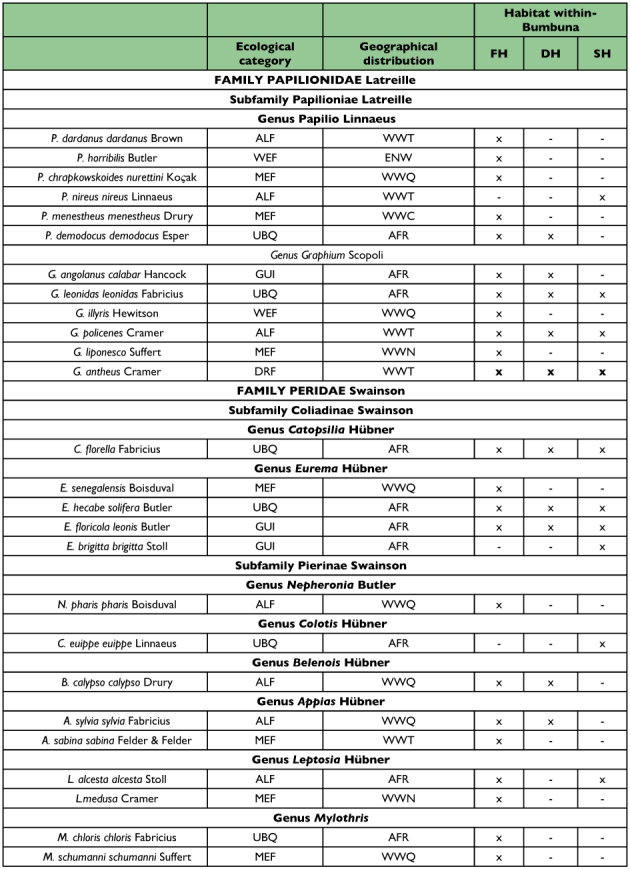
The preliminary check-list of butterfly species recorded from the Bumbuna Forest in the present study. Authorities, ecological category, geographic distribution (Belcastro and Larsen 2006) and habitat of capture within Bumbuna are given.
